# Rectal Hematoma Mimicking as a Gastrointestinal Stromal Tumor: An Atypical Endoscopic Ultrasound Finding

**DOI:** 10.7759/cureus.51898

**Published:** 2024-01-08

**Authors:** Mathew Thomas, Amrendra Mandal, Amanda Eisinger, Tessa Doolittle, Amitpal Nat

**Affiliations:** 1 Internal Medicine, State University of New York Upstate Medical University, Syracuse, USA; 2 Internal Medicine/Gastroenterology, State University of New York Upstate Medical University, Syracuse, USA

**Keywords:** rectal hematoma, gist, endoscopic ultrasound, colonoscopy, gastrointestinal bleeding

## Abstract

Acute gastrointestinal bleeding (GIB) represents a frequently encountered condition that prompts individuals to seek urgent medical attention at the emergency department, often leading to subsequent hospitalization. GIB can range from self-limited bleeding to hemorrhagic shock. Multiple etiologies contribute to the occurrence of GIB. In this report, we present the case of an 84-year-old male with multiple medical comorbidities admitted with hemodynamically stable lower GIB. Colonoscopy demonstrated a submucosal mass without evidence of bleeding. He subsequently underwent an endoscopic ultrasound (EUS) with sonographic findings concerning for a gastrointestinal stromal tumor. However, pathological analysis from both colonoscopy and EUS indicated the presence of blood, but no evidence of malignancy. A follow-up EUS performed two months later showed a complete resolution of the previously observed submucosal mass, suggesting that the initial evaluation was likely a hematoma that has resolved completely.

## Introduction

Acute gastrointestinal bleeding (GIB) poses a complex diagnostic and therapeutic challenge due to its diverse etiologies, ranging from benign conditions to life-threatening hemorrhagic events. GIB has been classified into upper and lower GIB in relation to the ligament of Treitz [1]. Lower GIB can arise from various sources, including diverticular disease, polyps, inflammatory bowel disease, vascular malformations, colorectal cancer, or anal fissures. A multidisciplinary approach involving gastroenterologists, surgeons, radiologists, and pathologists is often required to effectively manage GIB and optimize patient outcomes.

## Case presentation

Our patient is an 84-year-old male with a past medical history of stroke, diverticulitis, benign colorectal lesion status post colectomy, coronary artery disease, and aortic valve replacement (on clopidogrel), and he presented as a transfer from an outside hospital for further evaluation of rectal pain and hematochezia. The patient developed acute onset sharp non-radiating rectal pain for one day, associated with one episode of hematochezia. There was no history of abdominal pain, weight loss, nausea, or vomiting. 

**Figure 1:**
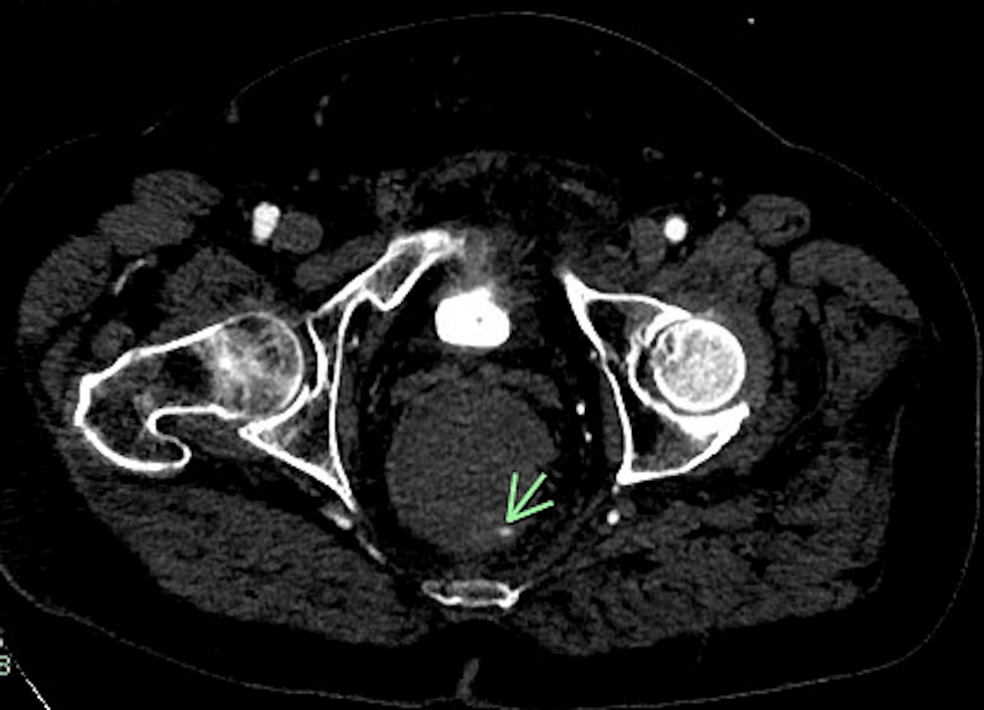
CT angiographic examination of the abdomen and pelvis demonstrating a focus of the intraluminal extravasation of the contrast along the left posterolateral aspect of the rectum.

In the emergency department, the patient was hemodynamically stable. Physical examination showed no abnormalities except for mild tenderness of the left iliac fossa. Laboratory evaluation revealed hemoglobin of 13.3 g/dL. Significant findings on computerized tomography angiogram (CTA) of the abdomen and pelvis included (a) intraluminal active vascular extravasation in the left posterolateral aspect of the rectum, and (b) a dilated rectum but rectal mass could not be excluded (Figure 1).

**Figure 2:**
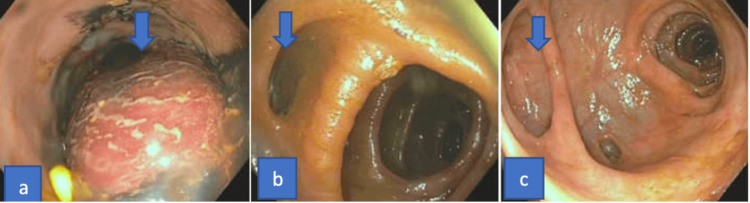
Colonoscopy demonstrating (a) a submucosal partially obstructing semi-circumferential mass in the rectum measuring 10 cm, about 5 cm from the anal verge, (b) diverticulosis in the sigmoid and descending colon, and (c) previous sigmoid anastomosis about 35 cm from the anal verge.

The initial differential diagnoses included a rectal mass (due to findings on CT), colorectal polyp (due to prior history), and diverticular bleed (due to the history of diverticulitis). Due to the CT findings of an active extravasation, interventional radiology was consulted, and the patient underwent coil anastomosis of the superior rectal artery. Gastroenterology was consulted for hemodynamically stable lower gastrointestinal bleeding, and the patient underwent esophagogastroduodenoscopy (EGD) and colonoscopy. EGD showed a medium-sized hiatal hernia and no evidence of bleeding. Colonoscopy findings were significant for (a) submucosal partially obstructing semi-circumferential mass in the rectum measuring 10 cm, about 5 cm from the anal verge, (b) diverticulosis in the sigmoid and descending colon, (c) previous sigmoid anastomosis about 35 cm from the anal verge, and (d) non-bleeding external and internal hemorrhoids. There was no evidence of intraluminal bleeding (Figure 2).

**Figure 3:**
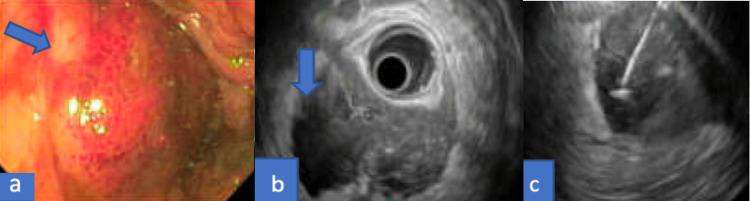
Endoscopic ultrasound (EUS) demonstrated a 4.7 cm x 3.8 cm non-circumferential mass located at the posterior rectal wall: (a) endoscopic image, (b) EUS image, (c) fine-needle aspiration (FNA) of the mass. EUS findings suggest a tumor originating from the muscularis propria with invasion into the perirectal fat, suggestive of a gastrointestinal stromal tumor (GIST).

As the colonoscopy showed a submucosal mass, an endoscopic ultrasound (EUS) was performed for further evaluation. This showed a 4.7 cm x 3.8 cm non-circumferential mass, located at the posterior rectal wall. There was sonographic evidence suggestive of a tumor originating from the muscularis propria with invasion into the perirectal fat. These sonographic findings were highly suspicious for gastrointestinal stromal tumor (GIST) of rectal origin (Figure 3).

**Figure 4:**
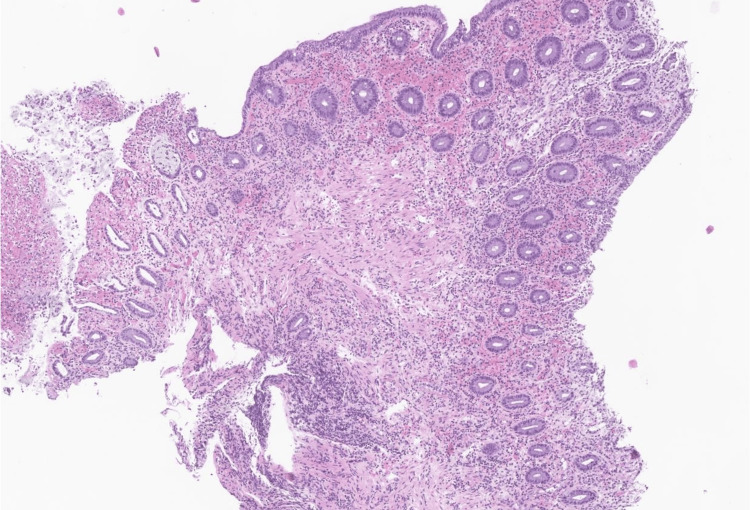
Rectal mass biopsy demonstrating benign colonic mucosa with patchy ulceration, moderate to severe activity, lamina propria hemorrhage, and features suggestive of ischemia. No adenomatous changes or malignancy is identified.

Fine-needle aspiration (FNA) was performed. The hospital course was complicated by significant drop in hemoglobin to 6.9- 9.2 g/dL (patient remained asymptomatic), for which the patient received a total of three units of blood transfusion during the entire hospital stay. Results of the colonoscopy with biopsy of the mass revealed benign colonic mucosa with patchy ulceration and lamina propria hemorrhage. There was no adenomatous changes or malignancy identified (Figure 4).

**Figure 5:**
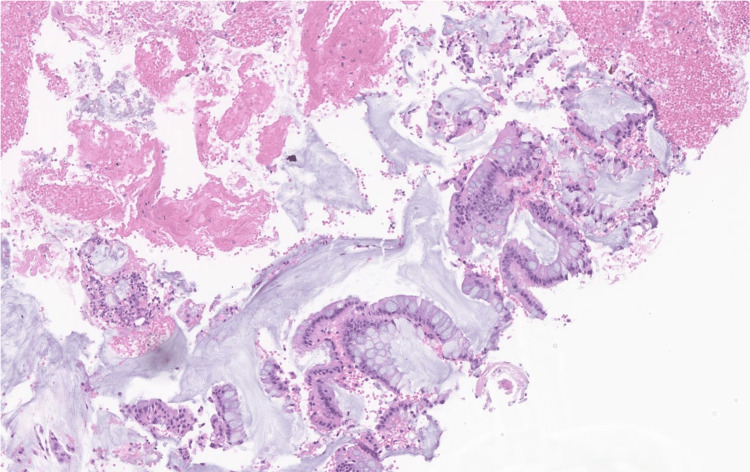
FNA of the submucosal mass demonstrating extensive blood with patchy detached superficial fragments of benign colonic epithelium with focal acute inflammation. No adenomatous change or malignancy is identified.

The results of the FNA demonstrated extensive blood with patchy detached superficial fragments of benign colonic epithelium with focal acute inflammation. There was no adenomatous change or malignancy identified. These pathology results were diagnostic of submucosal hematoma, rather than a solid tumor (Figure 5).

**Figure 6:**
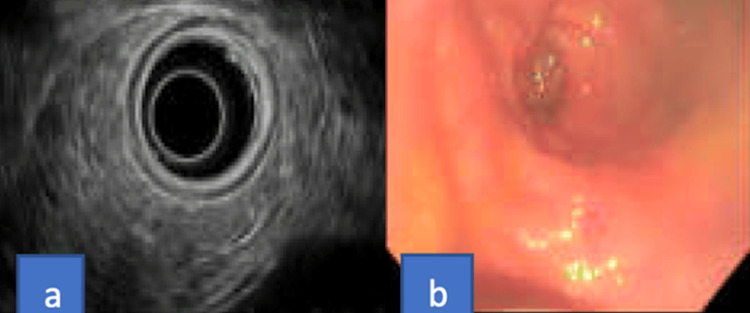
(a) Endosonographic image and (b) endoscopic image showing a normal rectum and recto-sigmoid with no evidence of submucosal mass.

The patient was discharged home in a stable condition, and underwent a repeat EUS in the outpatient setting, eight weeks from discharge. Repeat EUS demonstrated normal rectum and recto-sigmoid colon, and there was no evidence of submucosal mass (Figure 6). The large mass found on the previous exam was likely a hematoma, which was resolved completely.

## Discussion

Lower GIB is defined as bleeding arising from a source distal to the ligament of Treitz [1]. Lower GIB usually presents with hematochezia (passage of bright red, maroon, or blood clots per rectum). Blood originating from the right side of the colon is usually maroon-colored or mixed with stool, whereas blood originating from the left side of the colon is bright red in color [1]. For any patient presenting with GIB, initial patient assessment and hemodynamic resuscitation should be performed simultaneously [1]. Colonoscopy is the initial evaluation of choice in the diagnosis and treatment of hemodynamically stable acute lower GIB [1].

GIST is the most common mesenchymal neoplasm in the gastrointestinal tract [2]. GISTs arise from the interstitial cells of Cajal via mutation of the KIT proto-oncogene [3]. The most common sites of GIST involvement are the stomach (40-60%), small intestine (25-30%), colon/rectum (15%), and esophagus (<1%) [4]. Most GISTs originate from within the muscularis propria, whereas small lesions may originate from the muscularis mucosa [5,6]. On endoscopy, GIST appears as a submucosal mass with smooth margins, with normal overlying mucosa, and bulging into the gastrointestinal lumen. Central ulceration is occasionally seen [5]. On EUS, GISTs appear as hypoechoic, homogeneous lesions with well-defined margins, although they can rarely have irregular margins and ulcerations [5,6]. 

Rectal hematomas can clinically present as an intestinal obstruction or change in bowel habits [6]. As small as 30 ml of hematoma can cause symptomatic small bowel obstruction, but a larger amount of blood is needed to produce colonic obstruction [6]. In our patient, both colonoscopy with biopsy and EUS-guided FNA demonstrated blood and was negative for any malignancy, which raised our suspicion of rectal hematoma. Repeat EUS performed after two months showed a complete resolution of the mass, which confirmed our diagnosis. Our patient had a history of coronary artery disease and aortic valve replacement and was on clopidogrel, which could have precipitated the hematoma.

## Conclusions

GIB represents a diverse and challenging clinical entity that requires prompt recognition, accurate diagnosis, and management. The wide range of etiologies emphasizes the importance of a comprehensive evaluation to identify the underlying cause and guide the appropriate treatment decisions. GIST is a tumor of mesenchymal origin and can involve any part of the gastrointestinal tract. GIST has characteristic features on endoscopy and EUS. Our patient had features suggestive of GIST on endoscopy and EUS. However, pathological analysis indicated the presence of blood and no evidence of GIST. A follow-up EUS performed two months later showed a complete resolution of the previously observed mass, suggesting that the mass was likely a hematoma that has resolved completely.
